# Dimethyl Sulfoxide (DMSO) as a Potential Source of Interference in Research Related to Sulfur Metabolism—A Preliminary Study

**DOI:** 10.3390/antiox13050582

**Published:** 2024-05-09

**Authors:** Marta Kaczor-Kamińska, Kinga Kaszuba, Anna Bilska-Wilkosz, Małgorzata Iciek, Maria Wróbel, Kamil Kamiński

**Affiliations:** 1 Chair in Medical Biochemistry, Faculty of Medicine, Jagiellonian University Medical College, Kopernika 7 St., 31-034 Krakow, Poland; kinga.kaszuba@uj.edu.pl (K.K.); mbbilska@cyf-kr.edu.pl (A.B.-W.); malgorzata.iciek@uj.edu.pl (M.I.); mtk.wrobel@uj.edu.pl (M.W.); 2 Faculty of Chemistry, Jagiellonian University, Gronostajowa 2 St., 30-387 Krakow, Poland; kaminski@chemia.uj.edu.pl

**Keywords:** cystathionine γ-lyase, glutathione, 3-mercaptopyruvate sulfurtransferase, sulfane sulfur, sulfurtransferases, thiosulfate sulfurtransferase

## Abstract

Dimethyl sulfoxide (DMSO), an organosulfur compound, is widely used as the gold standard solvent in biological research. It is used in cell culture experiments and as a component of formulations in in vivo studies. Unfortunately, parameters related to sulfur metabolism are often not taken into account when using DMSO. Therefore, in this work we aim to show that the addition of DMSO to the culture medium (even in amounts commonly considered acceptable) alters some parameters of sulfur metabolism. For this study, we used three cell lines: a commercially available Caco-2 line (HTB-37, ATCC) and two lines created as part of our early studies (likewise previously described in the literature) to investigate the anomalies of sulfur metabolism in mucopolysaccharidosis. As the negative effects of DMSO on the cell membrane are well known, additional experiments with the partial loading of DMSO into polymerosomes (poly(ethylene glycol) methyl ether-block-poly(lactide-co-glycolide), PEG-PLGA) were performed to eliminate these potentially disruptive effects. The results show that DMSO is a source of interference in studies related to sulfur metabolism and that there are not just simple effects that can be corrected in the final result by subtracting control values, since complex synergisms are also observed.

## 1. Introduction

Dimethyl sulfoxide (DMSO)—an organosulfur compound ((CH_3_)_2_SO)—is a polar, aprotic solvent with low toxicity. The high polarity of DMSO allows it to dissolve many compounds that other solvents cannot [1], which is what makes DMSO a uniquely universal solvent. For this reason, it is widely used as a gold standard and the solvent of choice for biological research [2]. Many researchers have been using DMSO as a water-miscible vehicle for the delivery of molecules to cultured cells and as a component of formulations in in vivo experiments [3]. The first medical report concerning the usage of DMSO as a pharmacological agent was published in 1964 [4]. The United States Food and Drug Administration [5] and the International Council for Harmonization of Technical Requirements for Pharmaceuticals for Human Use [6] classified DMSO on the basis of its safety as class 3 (non-toxic). At present, two medicinal products (RIMSO-50 and Trametinib (trade name Mekinist)) containing DMSO as an active substance and formulation component, respectively, are approved for use by the FDA (https://www.fda.gov/drugs/development-approval-process-drugs/drug-approvals-and-databases; Approved Drug Products with Therapeutic Equivalence Evaluations, accessed on 15 March 2024). Nevertheless, the physiological and pharmacological properties and effects of DMSO are not yet fully understood.

Many scientific reports have been published indicating that this commonly used solvent affects various cellular processes (like the cell cycle, cell differentiation, lipid content, oxidative damage, apoptosis) and cell behaviors in different ways [7,8,9,10]. For instance, DMSO acts as a free radical scavenger at low concentrations (≤0.1%—nevertheless, the effect is strongly dependent on the cellular research model) [10,11,12]. DMSO traps free radical hydroxide (OH^·^), while its reduced metabolite—dimethyl sulfide (DMS)—traps free radical oxygen (O^·^) [13]. These DMSO capabilities may be partly responsible for some of the substance’s cryopreservative/cryoprotective and radioprotective properties [2,13]. DMSO is also known to have anti-inflammatory [3,10,12] and neuroprotective effects [3,10,14], and to interact strongly with phospholipids, which gives it useful properties as a membrane penetration-enhancing solvent for other low molecular weight compounds [1,2,3,15]. DMSO can easily pass through a biological membrane without permanently changing its structure, because of its hydrogen-bonding behavior, water affinity, ability to interchange with water in membranes, and ability to react with organic molecules [16,17]. Hence, DMSO has found application as a carrier/vehicle for drug administration and dissolving water-insoluble compounds in bioanalytical/biological screening tests [18].

During the drug discovery process, the effects of molecules/drugs properties and their structure–activity relationship (SAR) on biological assays are considered. There is an assumption that the SAR is built on interaction with the therapeutic target alone. If the SAR is affected by other factors, such as permeability through barriers, the solubility of the compound, and its chemical stability, then the above hypothesis will be wrong [19]. Among the issues related to the properties of the compounds, their solubility in aqueous buffers and DMSO is a major concern. The low solubility of the compound determines the lower range of its concentrations in screening assays. And this affects the activity manifested by the compound, which is not properly evaluated. It is also worth remembering that sometimes impurities present in the analyzed sample may be more soluble than the main ingredient. Thus, contamination, as well as the solvent itself, can be the reason for the observed activity or a measured property, leading to erroneous conclusions about SARs [19]. In the scientific literature, we can find reports in which the use of DMSO as a vehicle can alter the enzymatic activity of certain proteins [20,21,22,23,24,25,26]. In addition, Giugliarelli and others [27] showed that DMSO can induce protein aggregation and denaturation in cell cultures, and that the observed effect is neither protein nor cell type-specific. Therefore, it is important to be aware that the use of DMSO as a solvent for the substance of our interest (even in amounts generally considered acceptable) may provide an additional source of oxygen atoms, sulfur atoms, or monocarbon units for the various processes normally occurring in the cells [13]. However, these side-effects are often neglected [28,29].

There are limited data available on the effects of DMSO on the cellular sulfur metabolism. This is nevertheless surprising, because DMSO can be transformed into dimethyl sulfone (DMSO_2_, (CH_3_)_2_SO_2_) and sulfide (DMS, (CH_3_)_2_S) [8,11,17,29,30,31], which gives a distinctive and offensive garlic or oyster halitosis [32]. After the demethylation process, both of these compounds can be incorporated in the sulfur transformation pathways. The metabolism of sulfur in humans is complex and plays a central role in redox biochemistry (to read about cysteine metabolism in detail, please see [33,34,35,36,37,38]). For the studies included in this paper, we chose to investigate the effect of DMSO on the expression and activity of the following sulfurtransferases: thiosulfate sulfurtransferase (rhodanese, TST, EC: 2.8.1.1), 3-mercaptopyruvate sulfurtransferase (MPST, EC: 2.8.1.2), and cystathionine γ-lyase (CTH, EC: 4.4.1.1). All of these enzymes play an important role in the metabolism of L-cysteine [33,39,40]. TST transfers sulfur atoms from various donors (sulfane sulfur-containing compounds) to various acceptors [40,41]. Sulfane sulfur is defined as a sulfur atom covalently bonded to another sulfur atom and occurring in this system at a 0 or −1 oxidation state [33,40,42]. MPST catalyzes the transfer of the sulfur atom from 3-mercaptopyruvate to various acceptors, producing sulfane sulfur-containing compounds, (e.g., thiosulfate) or releases it as hydrogen sulfide [41,42]. CTH is also involved in sulfane sulfur generation in cells [33,39,40,42]. All enzymes mentioned above have sulfhydryl groups in their active sites [35,40]. These groups can be blocked by the oxidation process, leading to the inhibition of the enzyme’s activity. This blockade can be permanent or reversible (recovery by reaction of, i.a., glutathione/thioredoxine), depending on the oxidative capacity of the compound acting on the enzyme [35,37]. In order to gain insight into the antioxidant capacity of the cells tested, in our study we also controlled the level of sulfane sulfur, as well as the levels of low-molecular-weight, non-protein sulfur-containing compounds, such as the oxidized and reduced form of glutathione, cysteine ad cystine. The present publication is the result of the first observation of such a phenomenon and fills a research gap regarding the effect of DMSO on the non-oxidative pathway of L-cysteine metabolism.

The study was conducted on three different cell lines, including the commercially available Caco-2 line (HTB-37, ATCC) and two lines developed as part of our early studies on the abnormalities of sulfur metabolism in mucopolysaccharidosis (MPS) [38,43]. Caco-2 cells represent a well-established in vitro cell model that is used for drug permeability studies [44,45]. All the aforementioned cell lines have good characteristics in terms of the parameters studied [40,43] and, for practical reasons, can be treated as models for the studies presented in this paper. Moreover, lines derived from individuals with various diseases (cancer, metabolic disease) were deliberately selected to see whether it would be possible to identify any trend in the direction of changes in the parameters studied following the addition of DMSO. Moreover, as the negative effects of DMSO on the cell membrane are well known [1,2,3,15], in parallel to studies involving the direct addition of DMSO to the culture medium, a second method of administration was used—the partial insertion of DMSO into polymerosomes (poly(ethylene glycol) and poly(lactide-co-glycolide) methyl ether block, PEG-PLGA) aimed at eliminating these potentially destructive effects.

## 2. Materials and Methods

### 2.1. Preparation of Polymerosomes Partially Loaded with DMSO via the Film Rehydration Method

The polymerosome mixtures were prepared by dissolving the block PEG-PLGA (poly(ethylene glycol) methyl ether-block-poly(lactide-co-glycolide)) copolymer (molecular weight: PEG M_n_ 2000, PLGA M_n_ 4500, average M_n_ 6500 (total); lactide:glycolide ratio: 65:35; Sigma-Aldrich, Darmstadt, Germany) at a 10 mg/mL concentration in a chloroform solution (Sigma-Aldrich, Darmstadt, Germany). The chloroform was removed completely by evaporation. A thin and fine polymer film on the glass was obtained. The film was further dried under a constant flow of Nitrogen 3.5 (Messer Poland Ltd., Chorzow, Poland) for 3 min. Subsequently, the film was rehydrated with solutions of Dulbecco’s Modified Eagle’s Medium (DMEM, Sigma-Aldrich, Darmstadt, Germany) containing 10% DMSO (purity ≥ 99.9%; Sigma-Aldrich, Darmstadt, Germany). The vehicles were fabricated with a mix of the solution. To homogenize the sample, the suspension was subjected to sonication (UP50H, Hielscher, Teltow, Germany). The blank sample was prepared the same way, only it did not contain DMSO.

### 2.2. Characterization of Polymerosome Suspensions

Dynamic light scattering (DLS) was conducted using a Malvern Zetasizer Nano ZS Instrument (Malvern Instruments Ltd., Worcestershire, UK) equipped with a Peltier-controlled thermostatic cell holder. The laser wavelength was 633 nm and the scattering angle was 173°. Experimental diffusion coefficients, D, were measured at 25 °C. The Stokes–Einstein relationship D = k_b_T/3πηd_h_ was used to estimate the hydrodynamic diameter, d_h_. Here, k_b_ is the Boltzmann constant and η is the solvent viscosity. The size distribution of polymerosomes was determined using Zetasizer Software 7.12 (Malvern Instruments Ltd., Worcestershire, UK). The results were presented at number-based distributions.

### 2.3. Cell Culture

The experiments were carried out on human colorectal adenocarcinoma (Caco-2) cells, obtained from the American Type Culture Collection (ATCC: HTB-37) and two lines representing the murine cellular model of mucopolysaccharidosis, type IIIB: the WT (the control), and *Naglu*^−/−^ line (the line with mutation in gene encoding N-alpha-acetylglucosaminidase) [43]. The Caco-2 cells were cultured in DMEM supplemented with 20% (*v*/*v*) fetal bovine serum (FBS; Sigma-Aldrich, Darmstadt, Germany) and a 1% solution of penicillin and streptomycin (10,000 units/mL and 10,000 mg/mL (Sigma-Aldrich, Darmstadt, Germany), respectively). The WT and *Naglu*^−/−^ growth medium consists of DMEM with a 5% (*v*/*v*) FBS and 1% penicillin and streptomycin solution. The cultures were maintained at 37 °C, 95% humidity, and 5% CO_2_. Cells were cultured up to 80% confluence before being treated or harvested and stored at −80 °C.

### 2.4. Cell Homogenization

The pellets containing 3.5–5 × 10^6^ cells were suspended in 0.1 M phosphate buffer with a pH of 7.5 (Sigma-Aldrich, Darmstadt, Germany), maintaining the proportion of 1 million cells to 0.04 mL of the buffer, then sonicated for 3 × 5 s at 4 °C (Bandelin Sonoplus GM70, Berlin, Germany). Afterwards, they were centrifuged at 1600× *g* at 4 °C for 10 min and the supernatants were used to analyze the protein concentration, sulfane sulfur levels, and the activity of TST, MPST, and CTH.

In order to determine the low molecular weight sulfur-containing compounds by reversed phase high-performance liquid chromatography (RP-HPLC), the pellets were suspended in a 250 µL mixture consisting of 0.9% NaCl (Sigma-Aldrich, Darmstadt, Germany)/1 mM bathophenanthrolinedisulfonic acid disodium salt hydrate (BPDS, Sigma-Aldrich, Darmstadt, Germany)/70% perchloric acid (PCA, Polish Chemicals Reagents, Gliwice, Poland). The suspensions were then sonicated 3 × 5 s at 4 °C and centrifuged at 1600× *g* for 10 min (MPW-260R, MPW MED. INSTRUMENTS, Warsaw, Poland) to separate the sediment. The supernatants were collected and stored at −80 °C until RP-HPLC analyses.

### 2.5. The Crystal Violet Staining Assay

For the determination of cellular proliferation, the cells were seeded in triplicate on 24-well plates at a concentration of 5 × 10^4^ cell/well in a DMEM supplement, as reported above. Following 24 h of incubation, the culture medium was replaced with 500 µL medium with/without serum (depends on experiment, as a controls) or 500 µL of the appropriate medium containing various concentration of DMSO (1%, 2%, 3%, 4%, 5% and 10%), and then the cells were cultured for 24 h. Cell proliferation was examined using the modified crystal violet staining assay [46]. The absorbance was measured at 540 nm using an Epoch Microplate Spectrophotometer (BioTek Instruments Inc., Winooski, VT, USA). The relative cell viability (%) was expressed as the percentage of untreated control cells.

### 2.6. Enzyme Assays

#### 2.6.1. MPST Activity Assay

MPST activity was analyzed using the method of Valentine and Frankenfeld [47] with modifications described by Wróbel and others [48]. The mixture containing 250 µL of 0.12 M sodium phosphate buffer, pH 8.0, 50 µL of 0.5 M sodium sulfite (Sigma-Aldrich, Darmstadt, Germany), 50 µL of 0.15 M D,L-dithiothreitol (DTT, Sigma-Aldrich, Darmstadt, Germany), 50 µL of distilled water, 50 µL of the cell homogenate, and 50 µL of 0.1 M 3-mercaptopyruvate acid sodium salt (Sigma-Aldrich, Darmstadt, Germany), was incubated for 15 min at 37 °C. Then, 250 µL of 1.2 M PCA was added to stop the reaction and samples were centrifuged at 1600× *g* for 5 min. Next, 100 µL of supernatant was transferred to a solution consisting of 1200 µL of 0.12 M sodium phosphate buffer, pH 8.0, 100 µL of 0.1 M N-ethylmaleimide (NEM, Sigma-Aldrich, Darmstadt, Germany), and 50 µL of nicotinamide adenine dinucleotide reduced disodium salt hydrate (NADH, Sigma-Aldrich, Darmstadt, Germany). After equilibration at 37 °C, 2.5 µL of lactate dehydrogenase (LDH, 7 IU, Sigma-Aldrich, Darmstadt, Germany) was added, and the decrease in absorbance was measured at 340 nm. The enzyme activity was expressed as nmoles of pyruvate, which formed during 1 min incubation at 37 °C per 1 mg of protein.

#### 2.6.2. TST Activity Assay

The activity of TST was determined using Sörbo’s method [49] following a procedure used by Wróbel and others [48]. The reaction mixture consisted of 200 µL of 0.125 M sodium thiosulfate (Sigma-Aldrich, Darmstadt, Germany), 100 µL of 0.2 M potassium dihydrogen phosphate (Sigma-Aldrich, Darmstadt, Germany), 100 µL of homogenates, 100 µL of 38% formaldehyde (only blank sample; Polish Chemicals Reagents, Gliwice, Poland), and 100 µL of 0.25 M potassium cyanide (KCN, Sigma-Aldrich, Darmstadt, Germany). After 5 min incubation at room temperature, the following reagents were added: 100 µL of 38% formaldehyde (only to blank samples) and 500 µL of 0.2 M ferric nitrate reagent (Sigma-Aldrich, Darmstadt, Germany). The amount of thiocyanate formed during the reaction catalyzed with TST was measured colorimetrically at 460 nm. The enzyme units were defined as nmoles of SCN^−^ (thiocyanate), which formed during 1 min incubation per 1 mg of protein.

#### 2.6.3. CTH Activity Assay

The CTH activity was assayed by the method of Matsuo and Greenberg [50] with modifications by Wróbel and others [48]. The incubation mixture was prepared with 25 µL of 1.3 mM pyridoxal phosphate (Sigma-Aldrich, Darmstadt, Germany); 25 µL of 0.02 mM EDTA (Sigma-Aldrich, Darmstadt, Germany); 250 µL of 45 mM cystathionine (Sigma-Aldrich, Darmstadt, Germany) solution in 0.1 M phosphate buffer, pH 7.5 (2.5 mg of cystathionine per sample); and 75 µL of the cell homogenate. To achieve the final volume of 650 µL for each sample, a phosphate buffer of 0.1 M and pH 7.5, containing 0.05 mM of 2-mercaptoethanol (Sigma-Aldrich, Darmstadt, Germany) was added in appropriate amounts. The reaction ran for 30 min at 37 °C and was stopped by taking 125 µL of the incubation mixture into 25 µL of 1.2 M PCA. The samples were centrifuged at 1600× *g* for 10 min and then 25 µL of each supernatant was transferred to 625 µL of 0.194 mM NADH solution and kept at 37 °C. Controls (samples without 24 mM cystathionine) were prepared in the same manner. The measurement (absorbance at 340 nm) was conducted for 180 s; after 10 s, 25 µL of LDH (9.06 IU) was added. The difference between the initial value of absorbance (before LDH addition) and the lowest value (after LDH addition) corresponded to the amount of α-ketobutyrate formed in the reaction catalyzed with CTH. The enzyme activity was expressed as nmoles of α-ketobutyrate, which formed during a one-minute-incubation at 37 °C per 1 mg of protein.

### 2.7. The Sulfane Sulfur Level

The level of sulfane sulfur was assessed using the method of Wood [51]. This method allows for the colorimetric detection of the ferric thiocyanate complex ion and is based on a cyanolysis reaction. The incubation mixture for each sample consisted of 20 µL of 1 M ammonia solution (Polish Chemicals Reagents, Gliwice, Poland), 20 µL of cell homogenate, 740 µL of distilled water, and 100 µL of 0.5 M KCN, with a total volume of 880 µL. After a 45 min long incubation at room temperature, 20 µL of 38% formaldehyde and 40 µL of 0.2 M ferric nitrate reagent were added and the level of thiocyanate was estimated colorimetrically at 460 nm. The level of sulfane sulfur was expressed as nmoles of SCN^−^ per 1 mg of protein.

### 2.8. The Protein Content Determination

The protein content in the collected samples was determined by the method of Lowry and others [52], using the crystalline bovine serum albumin (BioShop Canada Inc., Burlington, ON, Canada) as a standard.

### 2.9. Determination of Concentration of Low-Molecular Weight Sulfur-Containing Compounds Using RP-HPLC

In order to determine the levels of the reduced (GSH) and oxidized (GSSG) glutathione, cysteine, and cystine, the RP-HPLC method of Dominic and others [53], with modifications described by Bronowicka-Adamska and others [54], was used. The samples were separated on a 4.6 mm × 250 mm Luna C18 (5 µm) column (Phenomenex, Warsaw, Poland) with a Phenomenex Security Guard column filled with the same packing material. The chromatographic system consisted of LC-10 Atvp Shimadzu Corp. pumps, four channel degassers, column oven, a Shimadzu SIL-10 Advp autosampler, and a Shimadzu Corp. SIL-10 SPD-M10Avp-diode array detector (Shim-Pol, A.M. Borzymowski, Warsaw, Poland); LabSolutions LC software was used to control the system operation and facilitate data collection. The standard curves were generated in the supernatant, which was obtained from cellular homogenates in the range from 13 to 75 nM of each compound per ml. All the standard curves generated for the analyte were linear in the investigated concertation range.

### 2.10. Determination of Total GSH Content In Vitro

Two 1 mM GSH (Sigma-Aldrich, Darmstadt, Germany) solutions were prepared: (A) in 10% (*v*/*v*) DMSO; (B) in pure water. The samples were left at room temperature for 48 h. The first measurement for both solutions was performed immediately, at time t = 0 min (the controls). Further measurements were recorded after 1, 3, 5, 7, 24, and 48 h. The determination of the GSH level is based on Ellman’s method [55], in which 5,5′-dithiobis-(2-nitrobenzoic acid) (DTNB, Sigma-Aldrich, Darmstadt, Germany) is reduced by -SH groups present in the sample, resulting in a product with an intense yellow color. The reaction mixture consisted of 850 µL of 0.2 M phosphate buffer, pH 8.2, 100 µL of 6 mM DTNB, and 50 µL of GSH solutions (A or B), respectively. Absorbance was measured spectrophotometrically against a blank containing DTNB and buffer at 412 nm. The total content of GSH was calculated from a standard curve prepared for 1 mM GSH. The results obtained for the DMSO-containing group were normalized to the GSH concentration determined in the non-DMSO-treated group and expressed as a percentage relative to the control (t = 0, 100%).

### 2.11. Total RNA Isolation

The extraction of the total RNA from the cells was performed using the TRI reagent/Trizol (Sigma-Aldrich, Darmstadt, Germany) following the protocol provided by the manufacturer. Next, extracted RNA was suspended in ribonuclease-free water (Thermo Fisher Scintific, Waltham, MA, USA) and its absorbance was measured at 260 nm for quantification. Afterwards, the purity of the obtained RNA samples was determined with a spectrophotometric analysis (A260 nm/A280 nm). In order to confirm the integrity of the isolated RNA, the separation of the 28 S and 18 S rRNA bands in 2.0% agarose-gel electrophoresis was performed. The samples were stored at −80 °C for further use.

### 2.12. Reverse Transcription of RNA

The reverse transcription of the total RNA from the cell samples was performed using a GoScript^TM^ Reverse Transcriptase Kit according to the manufacturer’s protocol (Promega, Madison, WI, USA). For the reaction, 2 µg of the total RNA were combined with water pretreated with diethylpyrocarbonate (DEPC-H_2_O, Thermo Fisher Scientific, Waltham, MA, USA) and with 1 µL of Oligo(dT)_15_ primer (0.5 µg/reaction, Thermo Fisher Scientific, Waltham, MA, USA) and incubated for 5 min at 70 °C. Then, a mixture with the following components was added: 4 µL of the GoScript^TM^ 5× concentrated reaction buffer (Promega, Madison, WI, USA), 3 µL of MgCl_2_ (final concentration 1.5–5 mM, Promega, Madison, WI, USA), 1 µL of deoxyribonucleotide triphosphates (dNTPs, 10 mM, Thermo Fisher Scientific, Waltham, MA, USA), 1 µL of Rnase inhibitor (20 U/µL, Thermo Fisher Scientific, Waltham, MA, USA), and 1 µL of GoScript^TM^ reverse transcriptase (Promega, Madison, WI, USA) (160 U/µL), giving a total volume of 20 µL. The samples were incubated for 5 min at 25 °C, then for 60 min at 42 °C, and finally for 15 min at 70 °C. The obtained solutions of complementary DNA (cDNA) were stored at 20 °C for further use.

### 2.13. Polymerase Chain Reaction (PCR)

The PCR was performed to analyze the expression of MPST, TST, CTH, and glyceraldehyde 3-phosphate dehydrogenase (GAPDH) genes. The gene encoding GAPDH (gene expressed normally in cells) was used as a reference (an international standard). The total volume of the cDNA amplification reaction was equal to 12.5 µL per sample and consisted of 1 µL of synthesized cDNA, 10 µM of each of gene-specific primer pair [40,43,56], 2 U/µL Taq DNA polymerase in 10 mM buffer Tris-HCl at pH 8.8 (Thermo Fisher Scientific, Waltham, MA, USA), and 10 mM of each dNTPs and DEPC-H_2_O. For the negative control, each reaction was also performed on the sample without cDNA. The reaction conditions and gene-specific primer sequences are published by Kaczor-Kamińska and others [40,43,56]. To ensure the accuracy of the results, each reaction was performed at least three times. All of the PCR products were analyzed in a 2.0% agarose gel stained with ethidium bromide (Sigma-Aldrich, Darmstadt, Germany), directly visualized under UV light, and photographed (ChemiDocTM MP Imaging system with Image Lab Software, version 6.0, Bio-Rad, Warsaw, Poland).

### 2.14. Statistical Analysis

All of the experiments were repeated at least three times. The results were presented as arithmetic means with standard deviations (SDs). The significance of the differences between controls and examined groups were calculated depending on whether the data met the assumptions of normality and variance homogeneity, either using the Student’s t-test (for data meeting the criteria of normal distribution) or the two-tailed Mann–Whitney U test (for data without normal distribution). The normal distribution of data was verified using the Shapiro–Wilks test. Differences with a *p* value < 0.05 were considered statistically significant. Statistical analyses were performed using Statsoft Statistica v.13 software (Tibco, Palo Alto, CA, USA).

## 3. Results

### 3.1. Effect of DMSO on the Polymer Vesicle Preparation and Loading

**Figure 1:**
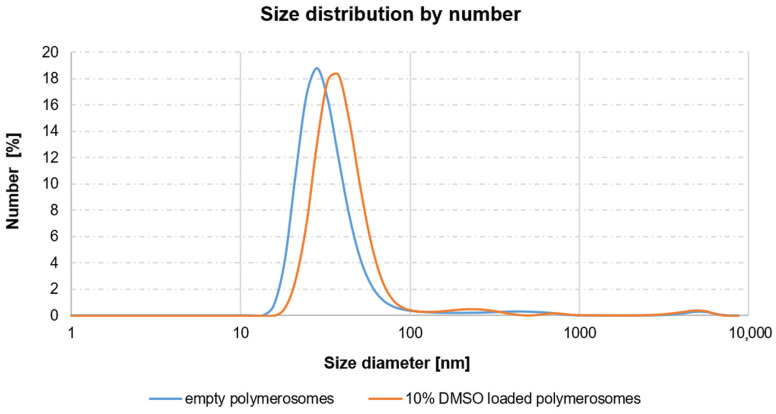
The analysis of polymerosomes performed with a DLS method.

The hydrodynamic size of the polymerosomes was measured using the Dynamic Light Scattering (DLS) method and a Malvern Zetasizer Nano ZS Instrument with an automatic adjustment of attenuation and measurement time. The empty polymerosomes and 10% DMSO-loaded polymerosomes were thoroughly characterized and the results are presented in Figure 1. The loading of DMSO did not significantly affect the size and aggregation of the polymerosomes, nor did it destabilize them (Figure 1).

### 3.2. Effect of DMSO on the Proliferation of Selected Cell Lines

**Figure 2:**
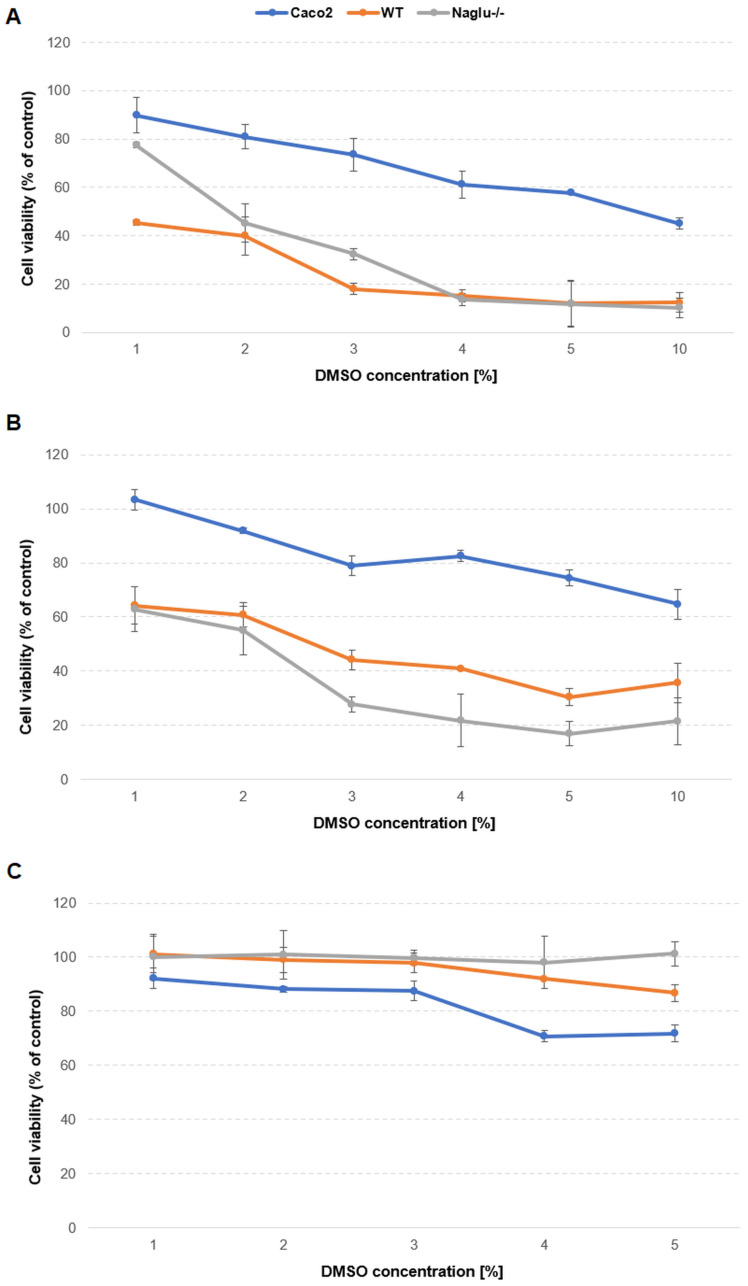
Cell proliferation dependence on concentration of DMSO administered directly (**A**) to the culture medium supplemented with serum and (**B**) into serum-free culture medium. (**C**) Concentration of DMSO-loaded polymerosomes administered directly into serum-free culture medium; exposure time—24 h: representative results for the selected cell lines (*n* = 3, experimental triplicate).

To examine the effect of DMSO (the concentrations range 1–10%) on the proliferation of human colorectal adenocarcinoma (Caco-2) cells and on the murine model of mucopolysaccharidosis, type IIIB, i.e., the WT (the control) and *Naglu*^−/−^ (the line with mutation in gene encoding N-alpha-acetylglucosaminidase) cell lines, the cells were treated with DMSO using three different ways of administration: (1) directly to the medium culture supplemented with serum (Figure 2A); (2) into the serum-free culture medium (Figure 2B); and (3) in DMSO-loaded polymerosomes administrated directly into the serum-free culture medium (Figure 2C). After 24 h, the cells were subjected to the crystal violet assay. The results obtained revealed a concentration-dependent effect of DMSO administrated directly to the culture medium (with and without serum) in all the selected cell lines (Figure 2A,B), whereby both MPS cell lines (the WT and *Naglu*^−/−^) showed a greater susceptibility to DMSO in a given concentration as compared to the Caco-2 cells. The highest DMSO concentration (10%) was cytotoxic to all the cell lines—the Caco-2, WT and *Naglu*^−/−^ cells. Proliferation decreased to about 45%, 10% and 10%, respectively, in the medium containing serum (Figure 2A), as well as about 65%, 35% and 20%, respectively, in the serum-free medium (Figure 2B). Conversely, when DMSO-loaded polymerosomes were administered to the culture medium of all selected cell lines, we observed only a mild decrease in cell viability with increasing DMSO concentration. With the application of polymerosomes containing 5% DMSO, the proliferation of the Caco-2 and WT cells decreased to about 70% and 90%, respectively, and in the case of the *Naglu*^−/−^ cells, it was maintained at the control level (Figure 2C). Based on the results obtained (Figure 2), concentrations of 1% DMSO for the WT and *Naglu*^−/−^ cell lines and 3% for the Caco-2 cell line were selected for further testing.

### 3.3. Effect of DMSO on the Level of Non-Protein Sulfur-Containing Compounds in the Selected Cell Lines

The level of sulfane sulfur-containing compounds was determined spectrophotometrically. The RP-HPLC method was used to determine changes in the levels of glutathione, L-cysteine, and L-cystine in the tested cell lines in the presence of DMSO. In both MPS cell lines, the level of sulfane sulfur-containing compounds and glutathione was altered after 24 h since DMSO was directly administrated to the culture medium supplemented with serum (Table 1). In the WT and *Naglu*^−/−^ cells, a reduction in sulfane sulfur level, by 18% and 23%, respectively, was observed. In the case of glutathione, a 32% reduction in its level was noted in the *Naglu*^−/−^ cells, while a 16% increase was observed in the WT cell line (Table 1). When DMSO was added directly to the serum-free culture medium, after 24 h, in the *Naglu*^−/−^ cell line, a 14% reduction in the level of sulfane sulfur-containing compounds was noted, while in the WT cell line, the cysteine level increased by 122% compared to levels determined in the corresponding control groups (Table 1). Similar results were obtained after modifying the way of introducing DMSO into the cells. Twenty-four hours after the administration of DMSO-loaded (1%) polymerosomes to the serum-free culture medium, a 21% decrease in sulfane sulfur-containing compounds level in the *Naglu*^−/−^ cells, as well as a decrease in glutathione levels by 35% (in the WT line), and 14% (in the *Naglu*^−/−^ line) compared to levels determined in their control groups, were observed (Table 1). Only one significant change was noted in the Caco2 cell line—a 12% increase in the level of sulfane sulfur-containing compounds after the addition of DMSO-loaded (3%) polymerosomes to the serum-free medium (Table 1). Therefore, based on the results, the human colorectal adenocarcinoma line appears to be the most unaffected by changes in non-protein thiol levels after DMSO administration, regardless of the method of its administration.

### 3.4. Effect of DMSO on Total Glutathione Level In Vitro

**Figure 3:**
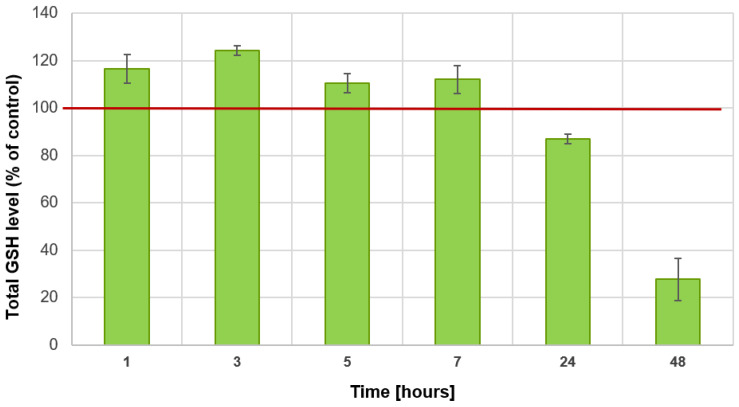
Effect of DMSO on the total GSH level—in vitro study (*n* = 3, experimental triplicate).

To investigate the direct effect of DMSO on the GSH level, an additional in vitro test was performed. From the results, it was found that the incubation of GSH in pure water at room temperature for 48 h did not result in significant changes in the amount of thiol groups—the GSH concentration was stable throughout the analysis, at 153.7 ± 5.7 nM. The results obtained for the second group, in which GSH was incubated with a 10% aqueous solution of DMSO (under the same conditions), revealed that initially (hours 1–7), the amount of available thiol groups increases—the greatest increase, of approx. 25%, was observed after 3 h (Figure 3). However, a progressive decrease in the number of thiol groups is already observed after one day. After 24 h, the number of thiol groups was approximately 13% lower, and after 48 h, by as much as 72% (Figure 3).

### 3.5. Effect of DMSO on the Expression of the Sulfurtransferases in the Selected Cell Lines

**Figure 4:**
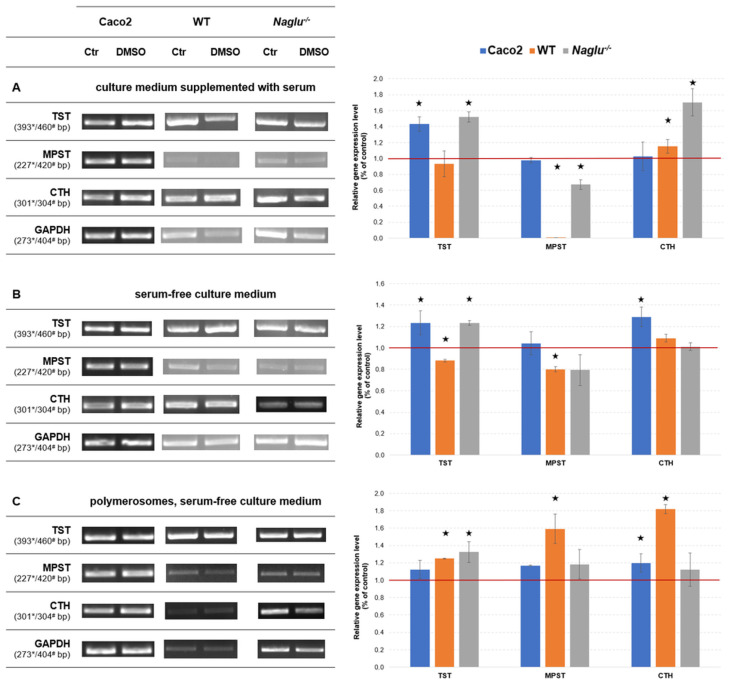
Effect of DMSO administration (**A**) to the culture medium supplemented with serum and (**B**) into the serum-free culture medium on sulfurtransferases expression level in the tested cell lines. (**C**) Effect of administration of DMSO-loaded polymerosomes into the serum-free culture medium on sulfurtransferases expression level in the tested cell lines. * PCR product length for the Caco-2 cell line; ^#^ PCR product length for the WT and *Naglu*^−/−^ cell lines. The methodology for the measurements is described in detail in our previous papers [40,43,56]. Densities of bands were normalized using the signal for the GAPDH gene. The results are representative and obtained from 3 tests. Statistical significance was set at * *p* < 0.05 (Mann–Whitney U test).

As shown in Figure 4, DMSO altered the expression levels of the sulfurtransferase genes studied, and the direction of these changes is similar in the vast majority of cases. Twenty-four hours after DMSO was added to the serum-containing medium, there was an increase in the expression level of the gene encoding TST in the Caco-2 (by 40%) and *Naglu*^−/−^ (by 50%) cells, and the gene encoding CTH in the WT (by 20%) and *Naglu*^−/−^ (by 70%) cells (Figure 4A) compared to the corresponding control groups. As for the gene encoding MPST, under these experimental conditions, a reduction in its expression level in the WT and *Naglu*^−/−^ cells by almost 100% and by 30%, respectively, was observed (Figure 4A). Similar results were obtained for an equivalent experiment conducted in serum-free culture medium (Figure 4B). In this case, we also observed an increase in the TST gene expression level in the Caco-2 and *Naglu*^−/−^ cells (by 20% in the both cases), as well as a 30% increase in the CTH gene expression level in the Caco-2 cell line compared to their control groups (Figure 4B). Moreover, twenty-four hours after the direct introduction of DMSO into the serum-free culture medium, a decrease in the expression level of the gene encoding MPST and TST, by 20% and 10%, respectively, was also observed in the WT cell line (Figure 4B). An interesting observation was noted when DMSO-loaded polymerosomes were introduced into the serum-free culture medium (Figure 4C). After changing the method of delivery of the compound to the cells, as in previous cases, a statistically significant increase in the expression level of the gene encoding TST in *Naglu*^−/−^ cells (by 30%) and the gene encoding CTH in the Caco-2 and WT cells, by 20% and 80%, respectively, were observed (Figure 4C). In this case, contrary to the results from the two previous experiments (Figure 4A,B), there was also an increase in expression levels for the gene encoding MPST and TST by 60% and 20%, respectively, in the WT cells (Figure 4C). In all other cases, the expression of the examined genes encoding the appropriate sulfurtransferases remained at the control levels (Figure 4A–C).

### 3.6. Effect of DMSO on the Activity of Sulfurtransferases in the Selected Cell Lines

**Figure 5:**
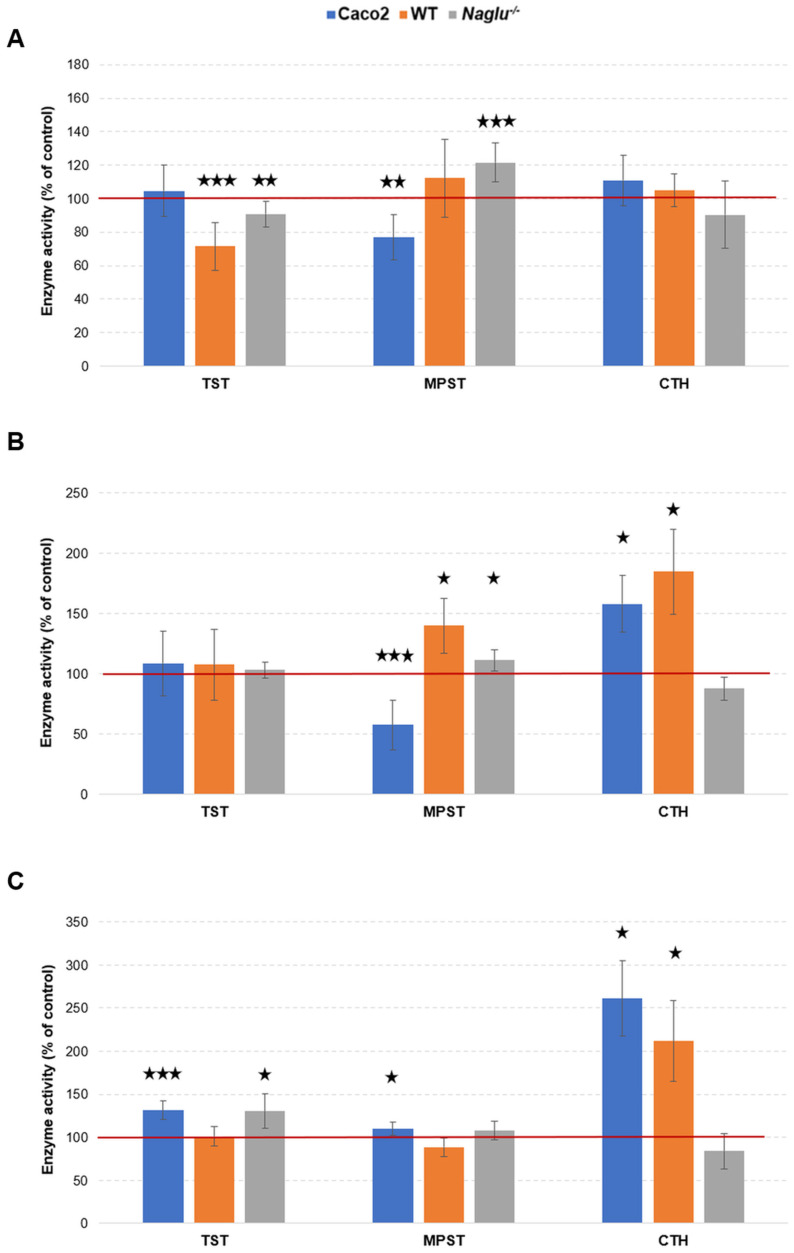
Effect of DMSO administration (**A**) to the culture medium supplemented with serum and (**B**) into the serum-free culture medium on sulfurtransferases activity. (**C**) Effect of administering DMSO-loaded polymerosomes into the serum-free culture medium on sulfurtransferases activity. The control values of TST, MPST, and CTH activities, determined after 24 h of culture of the Caco-2 cells, equaled, respectively, 92.5 ± 14.1; 508.1 ± 56.0; 2.6 ± 0.04 nmole/mg protein·min^−1^ (**A**); 35.0 ± 13.7; 413.2 ± 80.6; 4.4 ± 1.7 nmole/mg protein·min^−1^ (**B**); and 38.1 ± 3.2; 454.3 ± 35.3; 1.4 ± 0.7 nmole/mg protein·min^−1^ (**C**). In the WT cells, the control values of TST, MPST, and CTH activities are equaled, respectively, 34.0 ± 1.2; 215.9 ± 27.7; 1.4 ± 0.1 nmole/mg protein·min^−1^ (**A**); 24.7 ± 6.1; 39.5 ± 5.2; 0.9 ± 0.2 nmole/mg protein·min^−1^ (**B**); and 15.5 ± 1.9; 127.4 ± 13.3; 1.1 ± 0.01 nmole/mg protein·min^−1^ (**C**). In the *Naglu*^−/−^ cells, the control values of TST, MPST, and CTH activities are equaled, respectively, 17.2 ± 1.1; 170.6 ± 11.2; 2.0 ± 0.01; nmole/mg protein·min^−1^ (**A**); 12.3 ± 1.0; 110.7 ± 11.6; 1.1 ± 0.2 nmole/mg protein·min^−1^ (**B**); and 9.3 ± 1.6; 94.6 ± 8.7; 1.9 ± 0.3 nmole/mg protein·min^−1^ (**C**). The results are presented as arithmetic mean ± standard error of the mean (SEM) from three independent experiments. Statistical significance was set as follows: * *p* < 0.05; ** *p* < 0.01 and *** *p* < 0.001 (Student’s *t* test).

Based on the results shown in Figure 5, it can be observed that DMSO affects changes in the activity of sulfurtransferases under each of the conditions tested (Figure 5A–C). Twenty-four hours after DMSO was administered directly to the culture medium supplemented with serum, there was a statistically relevant reduction in TST activity in the WT and *Naglu*^−/−^ cells by about 29% and 9%, respectively, and a statistically relevant reduction in MPST activity in the Caco-2 cell line by about 23% (Figure 5A). On the other hand, in the *Naglu*^−/−^ cell line, contrary to the Caco-2 line, we noted an increase in MPST activity by 22% as compared to the MPST activity level determined in its control group (Figure 5A). The aforementioned direction of change in MPST activity is the same as that observed for cells of the Caco-2 and *Naglu*^−/−^ lines cultured in the serum-free culture medium (Figure 5A,B); decreases in MPST activity by 42% and increases by 12% were observed, respectively (Figure 5B). In addition, the administration of DMSO to the serum-free culture medium after 24 h resulted in an increase in MPST and CTH activity in cells of the WT line (by 40% and 85%, respectively) and Caco-2 line (CTH only by 58%). After the administration of DMSO-loaded polymerosomes to the serum-free medium, we also observed (Figure 5C) an increase in CTH activity in the Caco-2 and WT cell lines (by 161% and 112%, respectively), as well as MPST and TST activity in the Caco-2 cells (by 10% and 32%, respectively) and TST activity in the *Naglu*^−/−^ cell line (by 31%). In other cases, the activity of the tested sulfurtransferases remained at control levels (Figure 5A–C).

## 4. Discussion

### 4.1. DMSO Is Deleterious to Tested Cell Lines In Vitro

The result of this study showed that various cell types respond differently to DMSO and its effect is concentration-dependent. Cell viability was markedly reduced when treated with 1% (the WT and *Naglu*^−/−^ cell line) and 3% (the Caco-2 cells) DMSO, and its higher concentrations in the tested cell lines exerted an antiproliferative effect (Figure 2A,B). Scientific papers show various mechanisms by which DMSO affects cell proliferation, including, for example, cell cycle disruption, the disruption of cell differentiation, oxidative damage, or the induction of apoptosis [7,8,9,10]. However, extended research would be needed to determine this mechanism in our case. The non-toxic concentrations of DMSO reported in the literature are also controversial, varying greatly depending on the experimental conditions adopted, the exposure time, and the method of administration to the cells [58]. Some studies state that the critical concentration of DMSO dosed into cells should not exceed 1% [8,59], but one can find scientific literature where higher concentrations were used [16,60]. The presented results confirm that modifying the method of introducing the solvent into the cells (e.g., using DMSO-loaded polymerosomes) can allow the use of higher concentrations of the solvent without causing adverse effects on cell survival (Figure 2C). When DMSO (5%) was loaded into polymerosomes, they did not cause significant changes in cell viability in the tested lines: for the Caco-2, WT, and *Naglu*^−/−^ lines, the viable rates after 24 h were about 70%, 90%, and 100%, respectively (Figure 2C). This may support the hypothesis that one mechanism of DMSO cytotoxicity may be its direct effect on the physical properties of phospholipids in membranes [1,2,3,15].

### 4.2. DMSO Affects the Levels of Glutathione, Cystine, and Sulfane Sulfur-Containing Compounds

There are many studies showing the effects of DMSO on biological experimental systems [7], but there are relatively few papers documenting the interactions of DMSO with ubiquitous thiol antioxidants, such as glutathione and other sulfur-containing compounds, which are found at high levels in most cells [8,61]. Since it is feasible to react between the thiol and sulfoxide moieties of these molecules (as shown in Figure 3), in order to improve our knowledge of the bioactivity of DMSO, we tested our experimental models for the effect of DMSO on the level of non-protein, low-molecular-weight sulfur-containing compounds in the selected cellular research models. In accordance with the data collected in Table 1, it can be concluded that the effect of DMSO on GSH depends on the cell line, as well as the concentration of the compound used. The GSH level was reduced after the direct administration of 1% DMSO to the serum-enriched culture medium (the *Naglu*^−/−^ cells, about 32%), and after the addition of DMSO-loaded (1%) polymerosomes to the serum-free medium in the *Naglu*^−/−^ cells (about 14%) and WT cells (about 35%), compared to levels determined in appropriate control group (Table 1). An opposite effect—a statistically significant increase (by 16%) in the GSH level was observed after the direct addition of 1% DMSO to the serum-containing medium of the WT cells (Table 1). Nevertheless, our observations are in agreement with those already available in the literature, although the experiments were performed on different cell lines. In 2018, Dludla and others [8] showed that low doses of DMSO (<0.1%) increased the glutathione content in cells of the 3T3-L1 line, while higher doses of DMSO (>1%) significantly reduced its level in these cells. Homer and others [61] also observed that glutathione in human erythrocytes can be oxidized after adding DMSO in concentrations comparable to those used in cryopreservation, and this might have adverse effects on antioxidant balance in cells during that process. Under our experimental conditions, we did not observe a statistically significant increase in the level of the oxidized form of glutathione (GSSG, Table 1) in any case. The level of cysteine, a substrate for glutathione synthesis, remained beyond the limit of quantification for the method used (Table 1), but a statistically significant increase in the cystine level was observed in one case (Table 1) after the direct administration of 1% DMSO into the serum-free medium (the WT cells). Taking all of these aspects into account, it can be concluded that low-molecular-weight thiols such as glutathione and/or cysteine can be used to reduce DMSO [30,31], which may result in DMS formation in the experimental models tested. The route of the potential reduction of DMSO by thiols may follow several pathways, e.g., according to the mechanism proposed by Madesclaire in 1988 [62] (R1), but also by others (R1)–(R3) [8,11,30,31]:

(R1) $$RSH+R^{\prime }SH+(CH_{3}{)}_{2}SO\;\to \;RSSR^{\prime }+H_{2}O+(CH_{3}{)}_{2}S$$

(R2) $$RSH+(CH_{3}{)}_{2}SO\;\to \;RSOH+(CH_{3}{)}_{2}S\;$$

(R3) $$RSH+2\;(CH_{3}{)}_{2}SO\;\to \;RSO_{2}H+2\;(CH_{3}{)}_{2}S$$

According to these pathways, the pool of thiols and other sulfur-containing compounds that can reduce DMSO should decrease, which is reflected in the results presented in the paper (Table 1, Figure 3). The level of sulfane sulfur-containing compounds in the MPS cell lines (1% DMSO) was reduced regardless of whether the culture medium contained serum or not (the WT and *Naglu*^−/−^ cells), and regardless of how DMSO was introduced into the cells (the *Naglu*^−/−^ cells) (Table 1). In only one case, the opposite effect—a 12% increase in sulfane sulfur level (the Caco-2 cell line)—was observed after addition of DMSO-loaded (3%) polymerosomes to the serum-free culture medium. In the other two cases, the sulfane sulfur levels remained unchanged (Table 1). It can therefore be seen that, although the concentration of DMSO applied in the Caco-2 cell line was three times higher than that in the other two tested lines, it did not significantly alter the levels of sulfane sulfur or the levels of low-molecular-weight thiols compared to the respective control groups. Considering this fact, as well as the viability profiles determined for the cells of the Caco-2 line (Figure 2A–C), it can be concluded that these cells, compared to the WT and *Naglu*^−/−^ lines, show a different susceptibility to the same concentrations of applied DMSO and this is probably related to the different morphology and functionality of this line.

### 4.3. DMSO Causes Changes in the Non-Oxidative Metabolism of Sulfur

The results summarized in Figure 4 and Figure 5 show that the administration of DMSO to cells induces changes in the expression levels and activity of the sulfurtransferases tested (TST, MPST, and CTH), and that these changes in the cell lines taken for the study are complex. Changes in enzyme expression and activity (Figure 4 and Figure 5) may occur as a consequence of altered levels of glutathione and sulfane sulfur-containing compounds (Table 1), or these changes may be a direct cause. Any changes in the action of sulfurtransferases affect the bioavailability of cysteine and the availability of sulfane sulfur-containing compounds for various cellular processes.

Consider the DMSO-induced changes in the Caco-2 cell line. The addition of DMSO-loaded (3%) polymerosomes to the cells (serum-free culture medium) caused an increase in the levels of sulfane sulfur-containing compounds (Table 1), and this increase might be a reason for the increase in the cell’s demand for the enzymes involved in the formation of sulfur-donor molecules (mainly CTH) and their distribution (mainly MPST, TST)—hence the observed change associated with an increase in CTH expression (Figure 4C) and CTH, MPST, and TST activity (Figure 5C). In fact, when 3% DMSO was added directly into the serum-containing medium or serum-free medium, statistically significant changes in sulfane sulfur and glutathione levels in the Caco-2 cell line were not observed (Table 1). However, looking at the results for enzyme expression and activity (Figure 4A,B and Figure 5A,B), it can be seen here that the addition of DMSO results in the production of sulfane sulfur-containing compounds (expression and activity of CTH in serum-free medium increases, Figure 4B and Figure 5B), and there is an increase in the expression level of TST (Figure 4A,B), possibly enabling the activity of this enzyme to be maintained at control levels (no statistically significant change in TST activity, Figure 5A,B). As a result of the direct addition of 3% DMSO to the medium, MPST activity in the Caco-2 cells is reduced (Figure 5A,B). These two enzymes (CTH and MPST) transport sulfane sulfur atoms and form locally different structural systems (cyclic vs. linear persulfide, respectively) [40,63]; it may be that one of these transition modes is more favored than the other, depending on the methods of DMSO administration to the cells. Moreover, MPST has -SH groups in its active center, which can undergo reversible or irreversible oxidation to -SOH, -SO_2_H, or -SO_3_H [37], (R2) and (R3) either by the direct reaction of the enzyme with DMSO or indirect reaction with products (such as reactive oxygen species) of its metabolism. Unfortunately, too little data are available to indicate a direct cause—extended research is needed to clarify the mechanism of action of DMSO. However, the changes in CTH and/or MPST activity (Figure 5B) suggest that processes leading to the formation of various polysulfides (sulfane sulfur-containing compounds) were taking place in the analyzed cells. In addition, the process itself appeared to be effective under the proposed experimental conditions, as the determined sulfane sulfur level remained at control level (Table 1).

Consider the DMSO-induced changes in the two further cell lines. The *Naglu*^−/−^ cells (the line with metabolic disease), compared to cells of the WT line, have a documented lower antioxidant potential and a lower activity of sulfurtransferases enzymes [43]. Therefore, it could be expected that the observed effect of DMSO on sulfur metabolism in these lines would not be identical. However, the results obtained are overwhelmingly consistent (Table 1, Figure 4 and Figure 5). Analyzing the results that show the levels of sulfane sulfur-containing compounds, it can be seen that the results either showed no statistically significant change (the WT line, DMSO-loaded (1%) polymerosomes, serum-free culture medium) or the levels of sulfane sulfur were decreased after 24 h of exposure to DMSO (the *Naglu*^−/−^ cells in each case, the WT line—direct 1% DMSO administration to serum-containing and serum-free culture medium) (Table 1), and the same was true for glutathione levels—an increase in glutathione levels was only observed in one case (WT line, 1% DMSO, serum-enriched medium) (Table 1). The reduced glutathione levels might occur partly due to the redirection of L-cysteine into the pathway for the synthesis of sulfane sulfur-containing compounds, as evidenced by the observed increase in expression levels for the CTH gene in the WT (Figure 4A,C) and *Naglu*^−/−^ (Figure 4A) cell lines and in CTH activity (the WT cells, Figure 5B,C). The increase in MPST (in both cell lines, Figure 5A,B) and TST (the *Naglu*^−/−^ cells, Figure 5C) enzyme activity indicates that there was a ‘turnover’ of these compounds in the cell to maintain the homeostasis of sulfane sulfur-containing compounds (Table 1). The decrease in TST activity (in both WT and *Naglu*^−/−^ cells, Figure 5A) that was observed when DMSO was added directly to serum-enriched medium might suggest the active participation of this enzyme in ‘detoxification/neutralisation’ processes [40] (R1)–(R3) of DMSO molecules. This observation, together with the elevated glutathione level in the WT line (Table 1), may suggest that, under the proposed experimental conditions, L-cysteine was diverted into the glutathione synthesis pathway and was not available for the synthesis of sulfane sulfur-containing compounds, and that the available pool of the latter was reduced by direct or indirect reactions with DMSO and/or its metabolism products. Therefore, the results obtained (Table 1, Figure 4C and Figure 5C) indicate the consumption of poly/thiols for the reduction of DMSO, thus supporting the reaction mechanism (R1) proposed by Madesclaire [62] and others (R1)–(R3) [8,11,30,31].

The results regarding changes in expression may not appear consistent at first glance (Figure 4B vs. Figure 4C); however, when looking at the expression pattern for the enzymes tested, while taking into account how DMSO was introduced into the cells, it can be observed that the direct introduction of DMSO into the medium gives a consistent picture of the changes in the expression of the enzymes tested, regardless of whether serum was present in the medium or not (Figure 4A,B). Naturally, this expression pattern is slightly altered (TST and MPST expression in the WT line) when DMSO is administered to cells with polymerosomes (Figure 4C). However, the final biological effect related to the direction of changes in the enzymatic activity of the sulfurtransferases studied allows certain trends of change to be observed following the addition of DMSO (Figure 5).

The observed differences in the activities of the enzymes tested (Figure 5) seem to be dependent on the route of administration of the compound into the cells, and whether the culture medium contained serum. The activity of the tested sulfurtransferases, as well as the level of sulfane sulfur-containing compounds in the Caco-2, WT, and *Naglu*^−/−^ cells cultured with serum, was higher than in cells cultured for 24 h without serum (see description in Figure 5 and Table 1). Furthermore, there was also a greater amount of protein determined in cells cultured with serum compared to cells cultured in the serum-free medium over the same time period (24 h)—by up to approximately two times for the WT and *Naglu*^−/−^ lines. In addition, in the WT and *Naglu*^−/−^ cells, the level of reduced glutathione was also higher in cells cultured in the presence of serum compared to cultures grown without it (24 h, Table 1). Serum is a rich source of various amino acids, proteins, carbohydrates, lipids, hormones, vitamins, growth factors, growth inhibitors, minerals like Na^+^, K^+^, Zn^2+^, Fe^2+^, and trace elements [64]. This is because serum itself can stabilize or even boost sulfur metabolism in cells by providing them with compounds that, for example, enable the synthesis/regeneration of low-molecular-weight thiols and/or the regeneration of local damage by the reduction of oxidized groups in active centers in key enzymes in the pathway. In this work, we have shown that the addition of DMSO alone to a serum-free medium affects the antioxidant potential [37,65] of the cells tested (Figure 4B and Figure 5B, Table 1). Therefore, the addition of DMSO to the serum-containing medium may lead to the erroneous attribution of a positive effect to the compound of interest, rather than complex changes in cellular sulfur metabolism caused by the unintentional omission of additional sulfur-containing compound supplied with the solvent. For this reason, the presence of serum in the media during the experiment has many disadvantages and may lead to serious interpretive errors when analyzing the results, as these results depend on too many variables which are out of control. Additionally, DMSO can interact with serum proteins and/or macromolecules present in cells, and a lot of these interactions are still either unknown or far from being understood [9,27,66,67].

## 5. Conclusions

The presented observations are important, especially for researchers involved in studies related to sulfur metabolism and the detoxification processes of various xenobiotics. Even if many solvents are toxic to cells in vitro, we need them to introduce hydrophobic drugs into cells and conduct biological research and observations. However, it is important to be aware of the effect of the organosulfur solvent (DMSO) on the processes studied, even more so when a significant level of the solvent has been used to keep the molecule in the solution. The concentration of DMSO should always be given in scientific articles, which unfortunately is not always the case [28,29]. The impact of the solvent cannot be neglected, and the parameters related to sulfur metabolism should be under control. Any addition of DMSO can lead to changes in the antioxidant potential of cells by altering the expression and/or activity of sulfurtransferases (TST, MPST and CTH), and the levels of low-molecular-weight thiols and sulfane sulfur-containing compounds in the chosen cellular research model. Even if the study design provides a control group containing DMSO in an amount consistent with that present in the test group, the addition of DMSO to the culture medium (an amount generally considered acceptable) alters some parameters of sulfur metabolism and may be a source of interference by contributing to the erroneous attribution of the effect observed only to the analyzed compound. The error can be even greater when serum is present in addition to DMSO in the culture medium. The results presented in this paper indicate that the use of DMSO, even at the stage of sample preparation, should be seen as the introduction of an additional, external, independent factor capable of directly influencing the result obtained. Therefore, taking all the described facts into account, we believe that the scientific community should try to limit the use of DMSO in biological research, especially research related to sulfur metabolism. We recommend the use of other solvents or other methods to deliver difficult-to-solubilize compounds into cells (i.a., formulations based on micelles, liposomes, polymerosomes, or others available).

As the observed effects of DMSO on selected parameters of sulfur metabolism cannot be neglected, further extended studies will be required to elucidate the molecular mechanism(s) of action of DMSO and the extent of the interferences this compound causes in sulfur metabolism with short- and long-term exposure.

## Figures and Tables

**Table 1:** Sulfane sulfur and low-molecular-weight sulfur-containing compound levels in the selected cell lines.

Group		Sulfane Sulfur	Glutathione Reduced (GSH)	Glutathione Oxidized (GSSG)	Cysteine (CSH)	Cystine (CSSC)
		[nmole/mg Protein·min]	[nmole/mg Protein]			
	**culture medium supplemented with serum**					
**Caco2**	**Control**	349.88 ± 100.75	80.57 ± 7.9	<LOQ	0.08	<LOQ
	**3% DMSO**	300.63 ± 54.41	79.82 ± 7.0	<LOQ	<LOD	<LOQ
**WT**	**Control**	208.69 ± 6.16	134.45 ± 10.1	1.05 ± 0.1	<LOQ	<LOD
	**1% DMSO**	170.94 ± 15.33 ^###^	156.26 ± 7.2 *	0.25 ^a^	<LOQ	<LOD
***Naglu* ^−/−^**	**Control**	172.41 ± 15.46	119.97 ± 4.6	<LOQ	<LOQ	4.53 ± 0.1
	**1% DMSO**	132.32 ± 15.46 ^###^	81.06 ± 3.4 *	<LOQ	<LOQ	4.53 ^a^
	**serum-free culture medium**					
**Caco2**	**Control**	252.87 ± 106.31	76.47 ± 8.2	<LOQ	<LOQ	<LOQ
	**3% DMSO**	284.69 ± 72.99	83.36 ± 4.4	<LOQ	<LOQ	<LOQ
**WT**	**Control**	191.91 ± 21.92	109.44 ± 6.8	0.16 ± 0.1	<LOQ	2.21 ± 0.1
	**1% DMSO**	169.97 ± 11.45 ^#^	107.74 ± 13.5	<LOQ	<LOQ	4.90 ± 0.03 *
***Naglu* ^−/−^**	**Control**	129.04 ± 15.86	37.29 ± 6.6	<LOQ	<LOQ	<LOD
	**1% DMSO**	111.60 ± 10.13 ^#^	30.84 ± 1.3	<LOQ	<LOQ	<LOD
	**polymerosomes, serum-free culture medium**					
**Caco2**	**Control**	209.38 ± 28.48	82.12 ± 13.1	<LOQ	1.49 ^a^	<LOQ
	**3% DMSO**	233.56 ± 19.85 ^#^	86.18 ± 15.8	<LOQ	1.45 ^a^	<LOQ
**WT**	**Control**	159.29 ± 29.86	78.50 ± 4.7	<LOQ	<LOQ	<LOD
	**1% DMSO**	139.21 ± 30.80	50.75 ± 4.9 *	<LOQ	<LOQ	4.85 ^a^
***Naglu* ^−/−^**	**Control**	180.79 ± 30.43	53.90 ± 3.1	<LOQ	<LOQ	2.56 ^a^
	**1% DMSO**	143.42 ± 22.53 ^#^	46.45 ± 3.2 *	<LOQ	<LOQ	3.29 ± 0.02

The data are presented as the arithmetic mean with standard deviation. ^a^—the standard deviation was not calculated because of a low number of results; <LOD—lower than the limit of detection of the method; <LOQ—lower than the limit of quantification of the method. The limit of detection for glutathione (GSH) in the RP-HPLC method is equal to 0.01 (nmol·mL^−1^) and for oxidized form of glutathione (GSSG) it is equal to 0.1 (nmol·mL^−1^). The limit of quantification for GSH is 0.1 (nmol·mL^−1^) and GSSG is 1 (nmol·mL^−1^) [53]. The limit of detection for cysteine (CSH) was defined in the RP-HPLC method and is equal to 0.01 (nmol·mL^−1^) and for cystine (CSSC) it is equal to 0.1 (nmol·mL^−1^). The limit of quantification for CSH: 0.1 (nmol·mL^−1^) and the CSSC: 1 (nmol·mL^−1^) [53]. Total glutathione level in cells can be calculated according to the following formula: GSH + 2GSSG [57]. The Mann–Whitney U test was used to check for statistically significant differences between groups in the determined levels of glutathione, cysteine, and cystine (* *p* < 0.05, *n* = 3–4). The Student’s *t* test was used to verify differences in the determined levels of sulfane sulfur (^#^ *p* < 0.05; ^###^ *p* < 0.001; *n* = 3; number of replicates 10–15).

## Data Availability

The data presented in this study are available on request from the corresponding author.
